# Protective immunity against tuberculosis: what does it look like and how do we find it?

**DOI:** 10.1016/j.coi.2017.08.001

**Published:** 2017-10

**Authors:** Lu Huang, David G Russell

**Affiliations:** Microbiology and Immunology, College of Veterinary Medicine, Cornell University, Ithaca, NY 14853, United States

## Abstract

Progress towards the development of an effective vaccine against tuberculosis is hampered by the lack of correlative readouts of immune protection, coupled with our limited understanding of the immune mechanisms that determine disease progression versus containment. In this article we discuss the value of microbial readouts of bacterial fitness to probe the host immune environments and determine those host cell subsets that promote or control bacterial growth. Ultimately, we feel that these bacterial reporters will prove to be key in understanding the immune mechanisms underpinning disease outcome, and that this knowledge is critical to any program developing vaccines or immune-modulatory therapeutics as a means of controlling tuberculosis.

## Highlights

• An absence of immune correlates of protection is a barrier to vaccine development.
• The immune mechanisms behind tuberculosis progression are not understood.
• Fluorescent Mtb reporter strains identify permissive and controller host cells.
• Bacterial burden can be impacted by the magnitude of host cell population.
• Bacterial reporter strains offer new insights into host immune mechanisms.

**Current Opinion in Immunology** 2017, **48**:44–50

This review comes from a themed issue on **Host pathogens**

Edited by **Marc Pellegrini** and **Liz Hartland**

For a complete overview see the Issue and the Editorial

Available online 18th August 2017

**http://dx.doi.org/10.1016/j.coi.2017.08.001**

0952-7915/© 2017 The Authors. Published by Elsevier Ltd. This is an open access article under the CC BY-NC-ND license (http://creativecommons.org/licenses/by-nc-nd/4.0/).

## Introduction

The absence of measurable protection in the MV85A anti-TB vaccine trial population was reported in early 2013 [1]. This outcome forces us to acknowledge that we lack some of the most basic tools required for rational vaccine development. We do not have reliable correlates of protective immunity and we cannot actually define what constitutes a protective immune response capable of preventing disease progression in humans.

In this opinion piece we discuss the current status of immune correlates of protection, explore models of disease progression based on host cell phenotype, and propose that microbiological readouts of bacterial fitness represent the most rational pathway towards understanding the decision points that underpin the transition from latent TB infection (LTBI) to active disease.

## The disease

*Mycobacterium tuberculosis* (Mtb) is a human pathogen, and, as a species, we deal with the infection pretty well. Considering the penetrance of the pathogen in the human population, currently estimated at around 25% [2^••^], a remarkably small percentage of infected, immune-competent individuals (5–10%) go on to develop active disease. And while evolution will favor survival of the host, that selection pressure is survival beyond breeding age and not ‘complete’ protection. It is the sheer number of infected individuals, in combination with co-infection with HIV, that makes this pathogen such a burden to Global Health [3].

**Figure 1:**
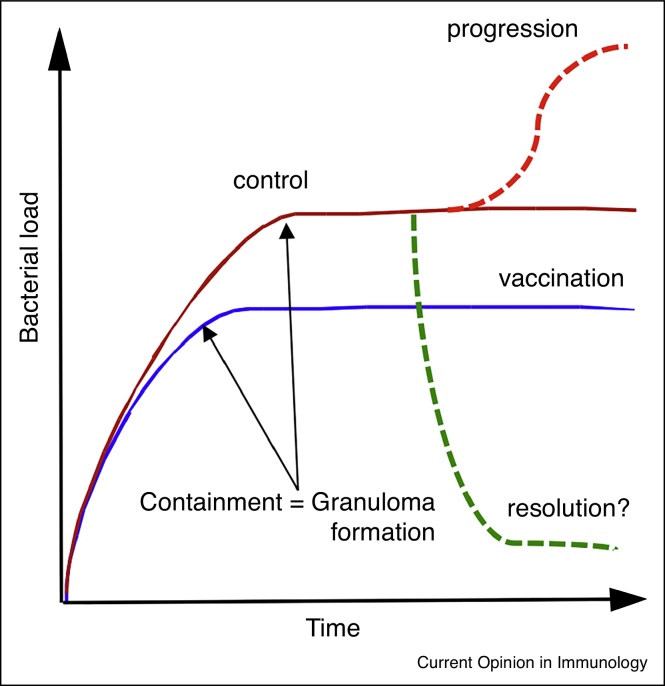
A schematic illustration of the potential outcomes of infection with Mtb. In most hosts Mtb exhibits rapid expansion of the bacterial burden over the first 3–4 weeks of infection. At this point the acquired immune response has developed and controls the bacterial burden at a subclinical level but is unable to clear the infection. In vaccinated hosts this transition to control of the bacterial burden is achieved at around a log fewer bacilli. While resolution of infection is possible theoretically most TB researchers do not believe it happens. Progression from latent disease to active disease appears to occur in the face of a robust systemic immune response that is Th1 dominant. While we have early indicators of disease progression, we have no immunological markers to detect vaccine-induced protection.

In Figure 1, we show a schematic representation of possible outcomes of Mtb infections in model hosts, discussed further by North and Jung [4]. In most instances the infection is marked by a rapid expansion of bacterial numbers prior to the establishment of an acquired immune response, which results in control of the bacterial burden without an apparent reduction in numbers. The induction of an immune response to Mtb through vaccination prior to challenge usually results in the set point for establishment of subclinical infection at approximately a log fewer infecting organisms. Given that neither previous infection nor vaccination protects against infection/re-infection we would argue that the most meaningful question in this disease continuum is the determination of those factors that facilitate transition from control to active disease within the context of a ‘protective’ immune response. Progression to active disease appears to be determined at the level of the individual granuloma, and is usually perceived as a loss of immune control but that remains an assumption. The granuloma is a complex cellular aggregate comprising predominantly of macrophages present as inflammatory macrophages, foamy macrophages and epitheliod macrophages, with T-cells present around the periphery [5]. The structure both contains the infection and provides haven to the infecting bacilli and it is this balance that determines progression or control [6, 7, 8, 9••, 10]. Increased bacterial growth could be due to increased permissiveness at the host cellular level that is independent of the pathways of immune-mediated control.

## Biomarkers of disease progression versus correlates of immune protection

Although often discussed together these two biomarkers categories are functionally very different. Recent publications on the identification of biomarkers predictive of disease progression are encouraging. Following the earlier, extensive transcriptional profiling of peripheral blood cells from individuals with active TB, a neutrophil-driven interferon-inducible gene signature was identified as indicative of a state of active disease [11, 12]. From that foundation, recent studies have tracked back to identify inflammatory biomarkers that can be detected prior to the development of active disease [13^•^]. These are predictors of disease development, but in reality they are readouts of early events in the progression to active disease, and are likely to be symptomatic rather than causal. As diagnostic indicators of disease progression that precede development of clinical disease, these biomarkers have tremendous potential in identifying individuals who ought to be placed on anti-tuberculosis therapy. Functionally, these are indicators of failed immunity, which are valuable diagnostic tools but are unlikely to provide functional insights into protective immune responses.

Having said that, our concept of useful markers of immune protection has been highly influenced by what we see in instances of failed immunity. IFN-γ-receptor deficiencies in humans, as with IFN-γ-deficiency in mice, renders them both exquisitely susceptible to Mtb infection and acute disease [14, 15, 16]. This has resulted in a self-fulfilling logic pathway that has led to the adoption of IFN-γ release assays (IGRA) as a surrogate for the acquisition of a protective immune response, which is not its primary function [17]. IFN-γ is clearly required for anti-bacterial responses but it appears insufficient for effective immune protection.

More recently other immune components have been studied as possible correlates for protection. Mice deficient in IL-17 and IL-17RA appear to be impaired in the recruitment of Th1 cells in a vaccine-induced protection model [18]. But data from different labs are contradictory on their role in protection and appear Mtb-strain-dependent [19]. NK cells are also implicated in control, and depletion of memory NK cells reduces vaccine-induced protection [20]. Finally γδ T cells are recruited to the lungs of Mtb-infected mice shortly after infection, and these cells can play a role in reducing viability of intracellular bacilli through perforin and granulysin-dependent mechanisms [21]. However, none of these candidates have been advanced to the point where they have the required robustness for assessing immune protection [22, 23, 24].

One of the assays that has gained increased traction recently is the mycobacterial growth inhibition assay (MGIA). It is attractive because it is a functional assay that measures the capacity of a cell population, usually from peripheral blood, to control Mtb growth upon co-culture. Multiple analyses by MGIA have been performed on whole blood and PBMCs of humans, mice and cattle, and none of which correlate with protective immune status [25]. The most robust outcome was a study on BCG vaccinated children where it was found that MGIA demonstrate Mtb control following primary but not secondary vaccination [26]. The repeated failure of MGIA performed on PBMCs to generate data correlative of protection would tend to indicate that the immune response in the peripheral circulation is an inadequate indicator of localized immune responses in infected tissue.

## The impact of host cell phenotype on bacterial burden

The phagocyte populations of the lung during early Mtb infection are extremely plastic. Ernst and colleagues conducted a detailed analysis of the different phagocytes infected with Mtb early following challenge [27, 28]. Mice were infected with GFP-expressing Mtb and the lung was harvested, and dissociated to generate single cell suspensions. They mapped the relative distribution of Mtb in the different phagocyte populations, including alveolar macrophages (AM), recruited interstitial macrophages (IM), monocytes (MO), dendritic cells (DC), and neutrophils (PMN). At day 14 the bacteria were equally distributed between AMs, myeloid DCs, and PMNs. The number of infected PMNs increased to day 21, and then declined sharply. At days 21 and 28 the majority of infecting bacteria resided in myeloid DCs.

These myeloid DCs are thought to differentiate from IMs, which show a dramatic increase in number during this early period of infection [27, 28]. Pamer's group showed that pulmonary infection with Mtb and *Aspergillus* drives recruitment of CCR2 and LyC6^−^ expressing inflammatory monocytes [29, 30]. Depletion of CCR2^+^ cells reduced transfer of the pathogen from the lung to the draining lymph nodes, abolished CD4^+^ T cell priming and impaired development of protective immunity. This is consistent with previous observations from Ernst's lab in murine tuberculosis infections where transport of Mtb to the draining lymph node was critical to the early priming of the immune response [31], and is in agreement with Skold and Behar's observation that monocytes recruited to the lung following Mtb infection acquire a mature DC phenotype [32]. These data all emphasize the extraordinary plasticity of the cell populations recruited to the early granuloma.

Interestingly, Leeman and colleagues demonstrated that depletion of AMs prior to infection with a lethal challenge dose of Mtb improved survival of the mice [33]. They then showed that specific depletion of activated macrophages was detrimental to the mice [34]. One interpretation of these data is that certain macrophages are required to provide a permissive niche for bacterial growth and that the depletion of classically activated (M1) macrophages reduces control of the infection. The idea that disease progression can be influenced both positively and negatively by the relative expansion of distinct subsets of phagocytes was supported by Antonelli and colleagues [35]. They treated mice intra-nasally with the Type 1 IFN inducer Poly (I:C) prior to infection with Mtb and found that this induced a marked increase in bacterial load in the lungs without impacting the Th1 immune response. Most significantly, this outcome was ablated when the experiments were conducted in CCR2-deficient mice indicating that the phenotype was dependent on the host cells recruited to the site of infection.

These studies all focus on initial stages of infection prior to and during development of the acquired immune response. However, phagocyte heterogeneity is also observed in established granulomas in non-human primates (NHP). Flynn and colleagues showed that TB granulomas in macaques contain many diverse types of phagocytes that express different markers such as Arg1, Arg2, iNOS and eNOS [36], proteins linked to the M1/M2 activation phenotype. These data were the basis for a model for granuloma progression driven by macrophage polarization developed by Kirschner and Flynn [9••, 37]. They argue that the ratio of M1/M2 polarized macrophages is predictive of granuloma outcome. The heterogeneity in phagocyte phenotype is also reflected in the relative balance of lymphocyte subsets in different granulomas in Mtb-infected macaques [38]. Finally, the manipulation of macrophage phenotypes in mice through the use of allergens, or the exploitation of different genetic backgrounds, impacts the ratio of M1/M2 type macrophage subsets and influences the bacterial burden [39, 40].

## Functional readouts of bacterial fitness

Many of these studies are based on the impact of perturbing the balance of phagocyte populations in the lung. What we do not know is the relative fitness or replication status of Mtb in these different phagocyte populations under ‘normal’ circumstances. We have investigated the changing transcriptional response in Mtb as it enters the macrophage and establishes an intracellular infection [41, 42, 43, 44]. One of the products of these studies has been the generation of reporter Mtb strains that express GFP under certain environmental cues relevant to the infection status of the macrophage [41, 45•, 46].

**Figure 2:**
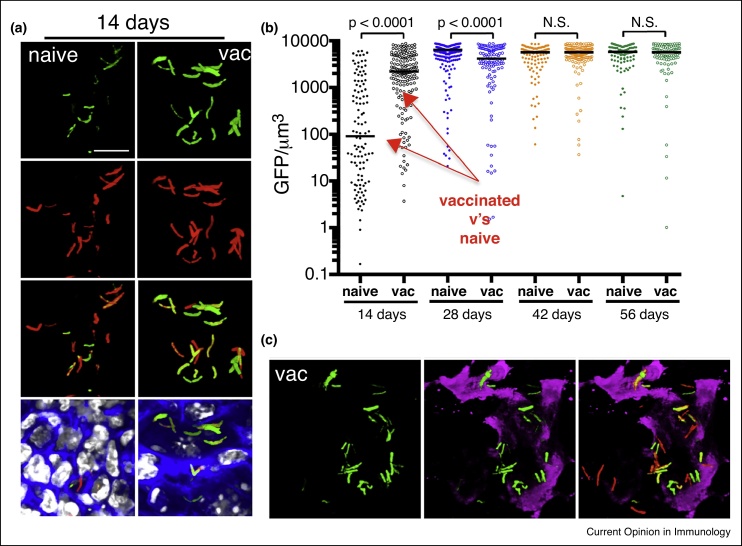
Demonstrating the application of the *hspX’::GFP* reporter strain in assessing and reporting on the localized induction of iNOS at the site of infection. PBS-immunized (naïve) and mice vaccinated with heat-killed *Mycobacterium tuberculosis* (vac) were infected with *hspX’*::GFP, *smyc’*::mCherry Erdman *M. tuberculosis* reporter strain. Fluorescence induction of the *hspX* promoter-dependent GFP is higher at 14 days in the vaccinated animals assessed by confocal microscopy of thick tissue sections **(a)**, that were scored subsequently by Volocity **(b)**. **(c)** The thick tissue sections were probed with antibodies against murine NOS2 (magenta) demonstrating the co-localization between GFP induction and NOS2 expression at the site(s) of infection. Data shown are detailed in Sukumar *et al.* [40].

We challenged naïve and vaccinated mice with fluorescent reporter strains of Mtb (Figure 2) [45^•^]. We demonstrated that induction of expression of *hspx’* promoter-driven GFP correlated directly with the presence of an acquired immune response, and localized to regions that stained positively with antibodies against NOS2 enzyme. Minimal label was observed in NOS2^−/−^ mice, indicating that the reporter was linked to production of NO. We also infected the mice with a replication reporter strain on Mtb with the single strand binding protein (SSB) fused to GFP and found that dots of SSB-GFP positivity were much more abundant early in naïve mice than in mice vaccinated previously with heat-killed Mtb. In addition, these replication indicators were expressed more frequently in Mtb in mice deficient in production of IFN-γ. These data demonstrate the validity of the strains to probe host cell phenotype at site of infection.

**Figure 3:**
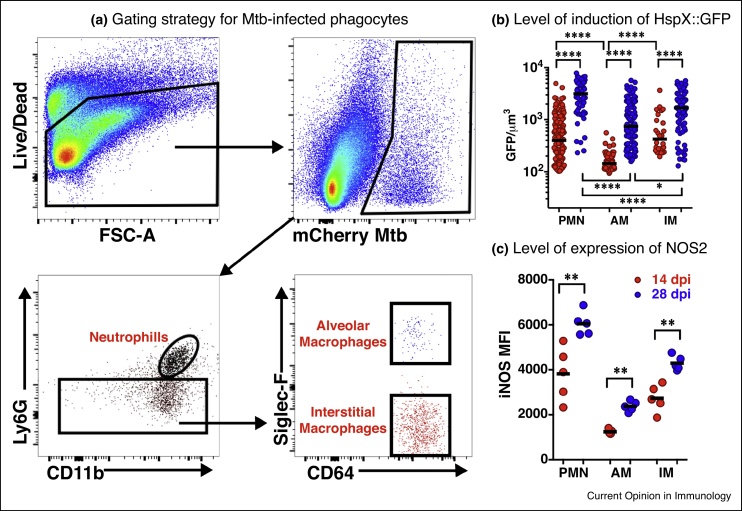
Examination of the bacterial stress reporter strain *hspX’*::GFP, *smyc’*::mCherry Erdman *Mycobacterium tuberculosis* at the level of different host phagocyte populations in murine lung infection model. **(a)** The flow cytometry gating strategy for the identification of *M. tuberculosis*-infected phagocyte subsets from infected mouse lung showing preliminary identification of alveolar macrophages, interstitial macrophages, and neutrophils. **(b)** The level of expression of NO-driven GFP under regulation of the *hspX* promoter identifying those phagocytes that induce the highest level of bacterial stress, and how the stress intensifies from 14 to 28 days post-infection. The levels of induction of expression of GFP indicate that the most stressful host cells appear to be neutrophils, and the least stressful, alveolar macrophages. **(c)** Labeling of the host cells with antibody against NOS2 demonstrates the direct correlation between expression levels of the host nitric oxide synthase, NOS2, and levels of expression of the bacterial stress response reporter *hspX’*::GFP (B).

We utilized fluorescent Mtb strains to probe the functionality of host cells in single cell suspensions generated from mice infected with the reporter bacteria (Figure 3) [47••, 48]. In initial studies utilizing bacterial fluorescence to identify infected host phagocytes we demonstrated that Mtb in immune-activated host phagocytes exhibited markedly higher levels of drug tolerance than those in resting phagocytes [47^••^]. Then using bacterial fitness reporter strains we found that Mtb in AMs showed relatively low expression of the bacterial stress readout *hspX’*::GFP [48] compared to those bacill in IM and neutrophils. The suggestion that AM represent a more permissive host cell environment to Mtb than the recruited IM is consistent with the previous study by Leemans and colleagues where the depletion of the AM population reduced bacterial burden and improved disease outcome in mice [33].

## Coupled metabolism of host and pathogen

Macrophage immunometabolism is a rapidly emerging area of research. In brief, M1 activated macrophages exhibit enhanced glycolysis whilst M2 macrophages show higher fatty acid oxidation [49, 50]. Tuberculosis infection is known to induce the Warburg effect in mouse lungs [51] and increased glycolysis is linked control of Mtb in human macrophages [52]. In contrast, active human disease is linked to dysfunctional lipid metabolism, accumulation of lipid droplets, and the formation of caseum from dead foamy macrophages [7, 53, 54, 55]. Moreover, a recent chemical screen against intracellular Mtb identified inhibitors of bacterial cholesterol breakdown as a critical nutrient acquisition pathway for this pathogen [56^•^]. Obviously, the metabolic status of Mtb infected pulmonary macrophages is more complicated and does not fit simply with our current knowledge of metabolism in M1/M2 macrophages. However, intranasal treatment of Mtb-infected mice with the TLR3 agonist poly (I:C) led to increased bacterial growth and accelerated disease progression [35]. Poly (I:C) is known to enhance triacylgyceride retention and reduce lipolysis in macrophages providing an environment that would, in theory, support enhanced mycobacterial growth [57]. These data suggest that progression of tuberculosis could be the product of expansion of a host cell type more supportive of bacterial growth through increased availability of nutrients, and that this expansion could occur independently of mechanisms of immune control.

Although this is not a quick fix, we believe that these bacterial fitness readouts will provide a mechanistic understanding of disease control and progression, and will ultimately identify those immune effector cells that need to be either expanded or contracted for vaccine-induced control of disease. We suspect strongly that disease outcome is due to the balance between permissive and controller host cells, and we are unlikely to understand this process until we appreciate the physiological basis of permissiveness, and not just control.

## Conflict of interest statement

Nothing declared.

## References and recommended reading

Papers of particular interest, published within the period of review, have been highlighted as:

• of special interest
•• of outstanding interest
